# Supplementary material for: Error Analysis and Modeling for an Absolute Capacitive Displacement Measuring System with High Accuracy and Long Range
Source: Sensors (Basel). 2019 Dec 4;19(24):5339. doi: 10.3390/s19245339 (PMC6994968; doi:10.3390/s19245339)
Supplement: Supplementary file 1 [file sensors-19-05339-s001.pdf]

sensors

MDPI

Supplementary

Error Analysis and Modeling for an Absolute Capacitive Displacement Measuring System with High Accuracy and Long Range

Dongdong Zhang [1,2,3], Li Lin [1,2,3,4,*] and Quanshui Zheng [1,2,3,4]

[1] State Key Laboratory of Tribology, Tsinghua University, Beijing 100084, China; zdd15@mails.tsinghua.edu.cn (D.Z.); zhengqs@tsinghua.edu.cn (Q.Z.)
[2] Department of Mechanical Engineering, Tsinghua University, Beijing 100084, China
[3] Center for Nano and Micro Mechanics, Tsinghua University, Beijing 100084, China
[4] Department of Engineering Mechanics, Tsinghua University, Beijing 100084, China
* Correspondence: linli@mail.tsinghua.edu.cn

S1. Modification of the Measurement Signal Model

1.1. Signal Functions Modified with Non-Orthogonality

As known from basic measuring principles, theoretically, $C_A(x)$ and $C_B(x)$ are orthogonal to $C_C(x)$ and $C_D(x)$, respectively, and $C_E(x)$ and $C_F(x)$ are orthogonal to $C_G(x)$ and $C_H(x)$, respectively. However, orthogonality can be affected by fabrication errors and installation errors. The signal functions for fine measurement and coarse measurement can be, respectively, rewritten as:

$$\begin{cases} C_A^{\alpha}(x) = +C_f(x+\phi_f+\alpha_f)+a \\ C_B^{\alpha}(x) = -C_f(x+\phi_f+\alpha_f)+b \\ C_C^{\alpha}(x) = C_C(x) \\ C_D^{\alpha}(x) = C_D(x) \end{cases} \tag{s1.1}$$

$$\begin{cases} C_E^{\alpha}(x) = +C_c(x+\phi_c+\alpha_c)+e \\ C_F^{\alpha}(x) = -C_c(x+\phi_c+\alpha_c)+f \\ C_G^{\alpha}(x) = C_G(x) \\ C_H^{\alpha}(x) = C_H(x) \end{cases} \tag{s1.2}$$

where α_f and α_c are very small constants.

1.2. Scaling Coefficient Functions

As described above, scaling coefficient functions were introduced to modify the signal model due to changes in amplitude and nonlinearity and can be expressed simply by polynomials. The scaling coefficient functions are described as follows:

$$f_p = \sum_{i=0}^{n} a_{pi} x^i, \qquad p = \mathrm{A, B, \ldots, H} \tag{s1.3}$$

In Equation (s1.3), n is the degree of the polynomials, and its value is determined according to the specific circumstances. The higher the degree of the polynomials is, the more complicated the variation it can express. The scaling coefficient function f_p is limited to $|f_p - 1| < \xi$ when $x \in [0, L]$, where ξ is a very small constant.

1.3. High-Frequency Noises

In practical applications, interference from electromagnetic signals and mechanical vibrations can lead to high-frequency noises that influence the measuring system's performance. For convenience, four of the same high-frequency noises, labeled N_f^m, were introduced into the

theoretical functions for fine measurement, and another four of the same high-frequency noises, labeled N_c^m, were introduced into the theoretical functions for coarse measurement. High-frequency noises have a variety of frequency components and can be described as:

$$\begin{cases} N_f^m = A_0^{Nf} + \sum_{n=1}^{\infty} A_n^{Nf} sin(n\omega_f x + \varphi_f) \\ N_c^m = A_0^{Nc} + \sum_{n=1}^{\infty} A_n^{Nc} sin(n\omega_c x + \varphi_c) \end{cases}. \quad \text{(s1.4)}$$

1.4. White Noises

In practical applications, white noise is ubiquitous in signals and affects the SNR; thus, it was introduced into the measuring signal model. For convenience, four different white noises of the same level, all labeled N_f, were introduced into the four theoretical functions for fine measurement. Four different white noises of the same level, labeled N_c, were also applied to the other four theoretical functions for coarse measurement. Both N_f and N_c were generated by the Gauss white noise equation.

S2. Signal Processing Method

S2.1. Differential Method

The differential method is defined as follows:

$$\begin{cases} y_{f1}^d = C_A' - C_B' \\ y_{f2}^d = C_C' - C_D' \\ y_{c1}^d = C_E' - C_F' \\ y_{c2}^d = C_G' - C_H' \end{cases}. \quad \text{(s2.1)}$$

According to Equation (10), Equation (s2.1) can be rewritten as:

$$\begin{cases} y_{f1}^d = (f_A + f_B)C_f(x + \phi_f + \alpha_f) + af_A - bf_B + N_f \\ y_{f2}^d = (f_C + f_D)C_f(x - T_f/4 + \phi_f) + cf_C - df_D + N_f \\ y_{c1}^d = (f_E + f_F)C_c(x + \phi_c + \alpha_c) + ef_E - ff_F + N_c \\ y_{c2}^d = (f_G + f_H)C_c(x - T_c/4 + \phi_c) + gf_G - hf_H + N_c \end{cases} \quad \text{(s2.2)}$$

where y_{f1}^d and y_{f2}^d are fine signals, and y_{c1}^d and y_{c2}^d are coarse signals. Simulated curves of fine signals and coarse signals are shown in Figure 4a.

In Equation (s2.2), take the expression of y_{f1}^d as an example for analysis. Because $|f_i - 1| < \xi$, $i = \mathrm{A}, \mathrm{B}$, that is, $f_i \approx 1, i = A, B$, the maximum variable signal of y_{f1}^d is:

$$\Delta y_{f1}^d = max(y_{f1}^d) - min(y_{f1}^d) \approx 4A_f. \quad \text{(s2.3)}$$

The other expressions of Equation (s2.2) can be analyzed using the same method, so $\Delta y_{f1}^d \approx \Delta y_{f2}^d \approx 4A_f$ and $\Delta y_{c1}^d \approx \Delta y_{c2}^d \approx 4A_c$. From Equation (s2.2), we can also obtain $N_{f1}^d = N_{f2}^d = \mathrm{N}_f$ and $N_{c1}^d = N_{c2}^d = N_c$.

The displacement resolution of fine signals and coarse signals is

$$\begin{cases} \delta_f = \frac{W_f}{4A_f/\sigma_{N_f}} \\ \delta_c = \frac{W_c}{4A_c/\sigma_{N_c}} \end{cases}. \quad \text{(s2.4)}$$

S2.2. Ratio Method

The ratio algorithm is defined as follows:

$$\begin{cases} y_{f1}^{r} = C_A'/C_B' \\ y_{f2}^{r} = C_C'/C_D' \\ y_{c1}^{r} = C_E'/C_F' \\ y_{c2}^{r} = C_G'/C_H' \end{cases}. \tag{s2.5}$$

According to Equation (10), Equation (s2.5) can be rewritten as:

$$\begin{cases} y_{f1}^{r} = \frac{f_A C_f(x+\phi_f+\alpha_f)+af_A+N_f+N_f^m}{-f_B C_f(x+\phi_f+\alpha_f)+bf_B+N_f+N_f^m} \\ y_{f2}^{r} = \frac{f_C C_f(x-T_f/4+\phi_f)+cf_C+N_f+N_f^m}{-f_D C_f(x-T_f/4+\phi_f)+df_D+N_f+N_f^m} \\ y_{c1}^{r} = \frac{f_E C_c(x+\phi_c+\alpha_c)+ef_E+N_c+N_c^m}{-f_F C_c(x+\phi_c+\alpha_c)+ff_f+N_c+N_c^m} \\ y_{c2}^{r} = \frac{f_G C_c(x-T_c/4+\phi_c)+gf_G+N_c+N_c^m}{-f_H C_c(x-T_c/4+\phi_c)+hf_H+N_c+N_c^m} \end{cases}. \tag{s2.6}$$

Similarly, y_{f1}^{r} and y_{f2}^{r} are called fine signals, and y_{c1}^{r} and y_{c2}^{r} are called coarse signals. Simulated curves of fine signals and coarse signals are shown in Figure 4b.

In Equation (s2.6), take the expression of y_{f1}^{r} as an example for analysis. Since $N_f + N_f^m \ll f_A C_f\left(x+\phi_f+\alpha_f\right) + af_A$ and $N_f + N_f^m \ll -f_B C_f\left(x+\phi_f+\alpha_f\right) + bf_B$, the term $N_f + N_f^m$ can be neglected. Besides this, there is $f_i \approx 1, i = A, B$, so:

$$y_{f1}^{r} \approx \frac{C_f(x+\phi_f+\alpha_f)+a}{-C_f(x+\phi_f+\alpha_f)+b}. \tag{s2.7}$$

The maximum variable signal of y_{f1}^{r} is:

$$\Delta y_{f1}^{r} = max\left(y_{f1}^{r}\right) - min\left(y_{f1}^{r}\right) \approx \frac{A_f+a}{-A_f+b} - \frac{-A_f+a}{A_f+b} = \frac{2(a+b)A_f}{(b+A_f)(b-A_f)}. \tag{s2.8}$$

The other expressions of Equation (s2.6) can be analyzed using the same method, so

$$\begin{cases} \Delta y_{f1}^{r} \approx \frac{2(a+b)A_f}{(b+A_f)(b-A_f)} \\ \Delta y_{f2}^{r} \approx \frac{2(c+d)A_f}{(d+A_f)(d-A_f)} \\ \Delta y_{c1}^{r} \approx \frac{2(e+f)A_f}{(f+A_f)(f-A_f)} \\ \Delta y_{c2}^{r} \approx \frac{2(g+h)A_f}{(h+A_f)(h-A_f)} \end{cases}. \tag{s2.9}$$

The noise in the fine signals and coarse signals cannot be directly obtained from Equation (s2.6). However, we can calculate it by taking the following approach:

$$\begin{cases} N_{f1}^{r} = y_{f1}^{r} - y_{f1}^{r0} \\ N_{f2}^{r} = y_{f2}^{r} - y_{f2}^{r0} \\ N_{c1}^{r} = y_{c1}^{r} - y_{c1}^{r0} \\ N_{c2}^{r} = y_{c2}^{r} - y_{c2}^{r0} \end{cases} \tag{s2.10}$$

where

$$\begin{cases} y_{f1}^{r0} = \frac{f_A C_A^{\alpha}}{f_B C_B^{\alpha}} \\ y_{f2}^{r0} = \frac{f_C C_C^{\alpha}}{f_D C_D^{\alpha}} \\ y_{c1}^{r0} = \frac{f_E C_E^{\alpha}}{f_F C_F^{\alpha}} \\ y_{c2}^{r0} = \frac{f_G C_G^{\alpha}}{f_H C_H^{\alpha}} \end{cases}. \tag{s2.11}$$

The displacement resolution of fine signals and coarse signals is:

$$\begin{cases} \delta_{fi} = \frac{W_f}{\Delta y_{fi}^r / \sigma_{N_{fi}^r}} \\ \delta_{ci} = \frac{W_c}{\Delta y_{ci}^r / \sigma_{N_{ci}^r}} \end{cases}, i = 1,2. \tag{s2.12}$$

S2.3. Differential–Ratio Method

The differential–ratio approach is defined as follows:

$$\begin{cases} y_{f1}^{dr} = \frac{C_A' - C_B'}{C_A' + C_B'} \\ y_{f2}^{dr} = \frac{C_C' - C_D'}{C_C' + C_D'} \\ y_{c1}^{dr} = \frac{C_E' - C_F'}{C_E' + C_F'} \\ y_{c2}^{dr} = \frac{C_G' - C_H'}{C_G' + C_H'} \end{cases}. \tag{s2.13}$$

Similarly, y_{f1}^{dr} and y_{f2}^{dr} are called fine signals, and y_{c1}^{dr} and y_{c2}^{dr} are called coarse signals. Simulated curves of fine signals and coarse signals are shown in Figure 4c.

According to Equation (10), Equation (s2.13) can be rewritten as:

$$\begin{cases} y_{f1}^{dr} = \frac{(f_A + f_B) C_f(x + \phi_f + \alpha_f) + a f_A - b f_B + N_f}{(f_A - f_B) C_f(x + \phi_f + \alpha_f) + a f_A + b f_B + N_f + 2N_f^m} \\ y_{f2}^{dr} = \frac{(f_C + f_D) C_f(x - T_f/4 + \phi_f) + c f_C - d f_D + N_f}{(f_C - f_D) C_f(x - T_f/4 + \phi_f) + c f_C + d f_D + N_f + 2N_f^m} \\ y_{c1}^{dr} = \frac{(f_E + f_F) C_c(x + \phi_c + \alpha_c) + e f_E - f f_F + N_c}{(f_E - f_F) C_c(x + \phi_c + \alpha_c) + e f_E + f f_F + N_c + 2N_c^m} \\ y_{c2}^{dr} = \frac{(f_G + f_H) C_c(x - T_c/4 + \phi_c) + g f_G - h f_H + N_c}{(f_G - f_H) C_c(x - T_c/4 + \phi_c) + g f_G + h f_H + N_c + 2N_c^m} \end{cases}. \tag{s2.14}$$

Using the same analytical method as for the ratio approach, we can obtain the maximum variable value of fine signals and coarse signals:

$$\begin{cases} \Delta y_{f1}^{dr} \approx \frac{4A_f}{a+b} \\ \Delta y_{f2}^{dr} \approx \frac{4A_f}{c+d} \\ \Delta y_{c1}^{dr} \approx \frac{4A_c}{e+f} \\ \Delta y_{c2}^{dr} \approx \frac{4A_c}{g+h} \end{cases}. \tag{s2.15}$$

The noise in the fine signals and coarse signals is calculated as follows:

$$\begin{cases} N_{f1}^{dr} = y_{f1}^{dr} - y_{f1}^{dr0} \\ N_{f2}^{dr} = y_{f2}^{dr} - y_{f2}^{dr0} \\ N_{c1}^{dr} = y_{c1}^{dr} - y_{c1}^{dr0} \\ N_{c2}^{dr} = y_{c2}^{dr} - y_{c2}^{dr0} \end{cases} \tag{s2.16}$$

where

$$\begin{cases} y_{f1}^{dr0} = \frac{f_A C_A^\alpha - f_B C_B^\alpha}{f_A C_A^\alpha + f_B C_B^\alpha} \\ y_{f2}^{dr0} = \frac{f_C C_C^\alpha - f_D C_D^\alpha}{f_C C_C^\alpha + f_D C_D^\alpha} \\ y_{c1}^{dr0} = \frac{f_E C_E^\alpha - f_F C_F^\alpha}{f_E C_E^\alpha + f_F C_F^\alpha} \\ y_{c2}^{dr0} = \frac{f_G C_G^\alpha - f_H C_H^\alpha}{f_G C_G^\alpha + f_H C_H^\alpha} \end{cases}. \tag{s2.17}$$

The displacement resolution of fine signals and coarse signals is:

$$\begin{cases} \delta_{fi} = \dfrac{W_{c1}}{\Delta y_{fi}^{dr} / \sigma_{N_{fi}^{dr}}} \\ \delta_{ci} = \dfrac{W_{c2}}{\Delta y_{ci}^{dr} / \sigma_{N_{ci}^{dr}}} \end{cases}, \quad i = 1,2. \tag{s2.18}$$

S3. Analysis of the Error Components of the Entire Experimental System

To understand different types of errors, we performed an analysis of the error components of the entire experimental system. In the experimental setup, a HEIDENHAIN-CERTO length gauge (H.) was used to calibrate the capacitive displacement system (CDS), and the characteristics of H. are shown in Table S1. The accuracy of H. is less than ±0.03 μm in a short-range measurement, and the repeatability/precision is also less than ±0.03 μm. As shown in Figure S1, there is uncertainty in the CDS, which refers to the difference between the H. displacement and the CDS displacement. This can be caused by two types of errors: (**1**) the first type of error is a connection error between the CDS and H., which is not a characteristic of the CDS itself; (**2**) the second type of error is a systematic error in the CDS itself, including the error analyzed in Section 6, which can be compensated for.

The first type of error certainly influences the accuracy of the CDS (Figure S1). However, such error is not a characteristic of the CDS itself. The error can be very small if we improve the accuracy of the calibration system. This type of error does not fall within the scope of our analysis. The second type of error, which is also called displacement calculation model error, is analyzed in Section 6. The uncertainty (±40 nm) in the displacement calculation model error was obtained according to the difference between the calibrated displacement and the calculated displacement. The uncertainty (±40 nm) is not a component of the first type of error. Thus, the uncertainty may be close to that of H.

Table S1. Characteristics of the HEIDENHAIN-CERTO length gauge.

System Accuracy (19 °C to 21 °C)	Short-Range Accuracy	Repeatability	Maximum Position Error Per Signal Period	Grating Period
less than ±0.1 μm; less than ±0.05 μm[1]	less than ±0.03 μm	less than ±0.03 μm	less than ±0.02 μm	4 μm

[1] After compensating for linear length error.

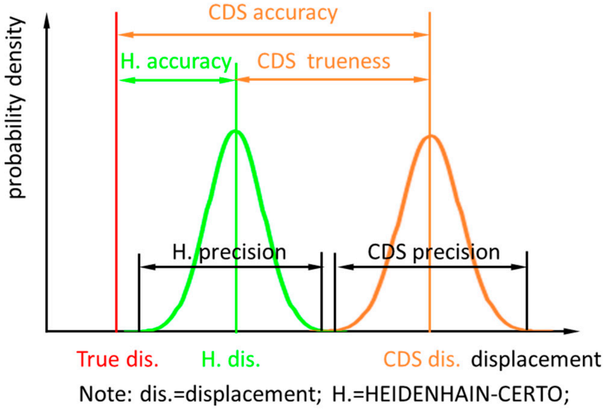

Figure S1. Accuracy of the entire experimental system.
